# Calibration and segmentation of skin areas in hyperspectral imaging for the needs of dermatology

**DOI:** 10.1186/1475-925X-13-113

**Published:** 2014-08-08

**Authors:** Robert Koprowski, Sławomir Wilczyński, Zygmunt Wróbel, Barbara Błońska-Fajfrowska

**Affiliations:** Department of Biomedical Computer Systems, Faculty of Computer Science and Materials Science, University of Silesia, Institute of Computer Science, ul, Będzińska 39, Sosnowiec, 41-200 Poland; Department of Basic Biomedical Science, School of Pharmacy, Medical University of Silesia in Katowice, ul, Kasztanowa 3, Sosnowiec, 41-200 Poland

**Keywords:** Hyperspectral imaging, Image processing, Measurement automation, Segmentation, Dermatology, Calibration

## Abstract

**Introduction:**

Among the currently known imaging methods, there exists hyperspectral imaging. This imaging fills the gap in visible light imaging with conventional, known devices that use classical CCDs. A major problem in the study of the skin is its segmentation and proper calibration of the results obtained. For this purpose, a dedicated automatic image analysis algorithm is proposed by the paper’s authors.

**Material and method:**

The developed algorithm was tested on data acquired with the Specim camera. Images were related to different body areas of healthy patients. The resulting data were anonymized and stored in the output format, source dat (ENVI File) and raw. The frequency *λ* of the data obtained ranged from 397 to 1030 nm. Each image was recorded every 0.79 nm, which in total gave 800 2D images for each subject. A total of 36'000 2D images in dat format and the same number of images in the raw format were obtained for 45 full hyperspectral measurement sessions. As part of the paper, an image analysis algorithm using known analysis methods as well as new ones developed by the authors was proposed. Among others, filtration with a median filter, the Canny filter, conditional opening and closing operations and spectral analysis were used. The algorithm was implemented in Matlab and C and is used in practice.

**Results:**

The proposed method enables accurate segmentation for 36’000 measured 2D images at the level of 7.8%. Segmentation is carried out fully automatically based on the reference ray spectrum. In addition, brightness calibration of individual 2D images is performed for the subsequent wavelengths. For a few segmented areas, the analysis time using Intel Core i5 CPU RAM M460@2.5GHz 4GB does not exceed 10 s.

**Conclusions:**

The obtained results confirm the usefulness of the applied method for image analysis and processing in dermatological practice. In particular, it is useful in the quantitative evaluation of skin lesions. Such analysis can be performed fully automatically without operator’s intervention.

## Introduction

Hyperspectral imaging is now one of the most developed methods of visible light imaging [1, 2]. It enables to acquire data of an object in any spectral range set in the camera. This method is entirely non-contact and non-invasive, and measurements can be carried out remotely. With these advantages, hyperspectral cameras are used in many fields of technology and medicine [3–7]. In particular, they are used in dermatology. The issue of dermatological research concerns spectral skin analysis in almost all cases. This analysis is usually carried out on the basis of data recorded by a camera and saved in dat or raw format, or another one dependent on the type of camera used [8, 9]. These data are further analysed in the software provided directly by the camera manufacturer or, less often, in another software. The latter option is rarely used as there is a need to meet the following requirements:

- the possibility to analyse large amounts of data - analysis of files that are a few or tens of gigabytes in size,
- the need to correctly read data, most often in dat or raw format,
- calibration of the results obtained.

A large amount of data prevents simple applications from analysing such large images - a typical file size is several gigabytes, as mentioned earlier. The data in the file are saved in 32 or 16-bit format depending on its type, dat or raw. Consequently, this leads to differences in the file volume and the quality of the resultant image. Regardless of the format of data record, a sequence of hyperspectral images requires calibration. Due to the different brightness of lighting (from the definition of linear spectral characteristics), calibration of images in the range from black (0% brightness) to white (100% brightness) is necessary. This goal is achieved in different ways. In the simplest case, it is a white stripe, pattern, taken as 100% brightness, that is placed next to the imaged skin. Subsequent images are calibrated with respect to it. The calibration stage ends with suitable linear adjustment of brightness levels. Placing a white stripe pattern at the top of the image is sometimes used and recommended by Specim Company and LOT-Quantum Design Company. Automatic its analysis here is not done in a dedicated program ENVI. Therefore, it is not possible implementation of automatic calibration.

The second major area of problems related to hyperspectral imaging is image analysis. On the one hand, typical image analysis and processing in visible light can be applied. On the other hand, the acquisition of a separate image for each length of the spectrum gives much wider possibilities of analysis. Typical tasks, most commonly used in dermatology, include segmentation of skin areas. In general, morphological methods [10–15], statistical methods [16–18] and algorithms profiled to selected applications are known from hyperspectral imaging. Among the morphological methods, there are classical approaches [1, 13, 14] and those profiled to the analysis of an image sequence [12, 15]. In the statistical methods, there dominates texture analysis [17–21] used as a set of features for classification and recognition. Profiled algorithms have been applied so far to face recognition [22], analysis of skin areas [23], and others [24–27]. These methods are mainly related to segmentation of specific objects [28]. On the basis of segmented objects, their morphometric measurements or their texture analysis are carried out [18]. However, for skin areas, an important feature is their location in the image, which is vital, inter alia, in the assessment of efficacy and safety of treatments in aesthetic medicine [29–31]. A description of this type of automatic method preceded by automatic calibration is presented below.

## Material

The study used images of human healthy skin obtained with the Specim camera PFD-V10E. The measured body areas were lit using a typical lamp with flat spectral characteristics in the required range (based on HgAr emission for the VNIR spectral range). The images were obtained retrospectively during routine medical (dermatological) examinations, performed in accordance with the Declaration of Helsinki. In connection with the described algorithm, no research or experiments were carried out on humans. The acquired data were anonymised and stored in the output format, source dat (ENVI File) and raw. The frequency λ of the data obtained ranged from 397 to 1030 nm. Each image was recorded every 0.79 nm, which in total gave 800 2D images for each patient. The resolution *M* × *N* (number of rows and columns) of each image for the selected frequency was varied depending on the scan area. It was usually *M* × *N* = 899 × 1312 pixels. The number of rows *N* was changed most often and was in the range *N*∈(15,899). Regardless of the distance of the camera from the object and selected focusing parameters, one pixel covered a square area in the range of 130 μm × 130 μm. A total of 36’000 2D images were obtained for 45 hyperspectral images. These images were subjected to further analysis.

## Method

Hyperspectral image analysis method is associated with three stages:

- Pre-processing of images in which filtration is the main element,
- Calibration linked to the automatic recognition of the pattern position,
- Processing of images enabling proper segmentation of the skin areas.

Details of these three stages are described below.

### Pre-processing

**Figure 1:**
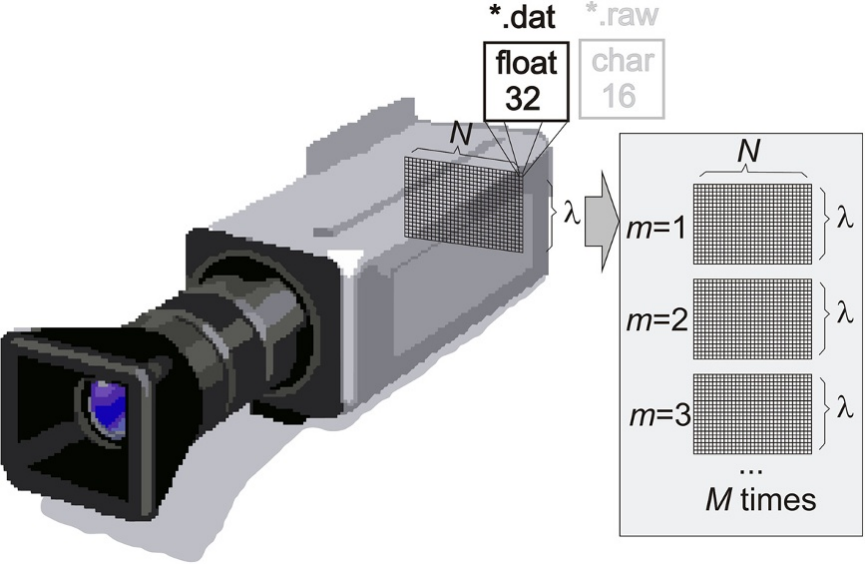
Block diagram of the acquisition and organization of *.dat and *.raw *data. Data are recorded at the same time for each image line for all wavelengths *λ*. In this way, to create a two-dimensional image *M* × *N* for a particular wavelength *λ*, every *M*-th row should be read in the *.dat or *.raw file. This process is carried out at the stage of pre-processing and data acquisition.

Image pre-processing concerns correct reading and interpretation of the data recorded by the camera PFD-V10E in dat and raw format. This camera records information for each line (each row) *N* and at the same time registers a full spectral range. In this case, *λ*∈(397, 1030) nm is equivalent to the adopted spectral distance with the registration of 800 lines. This process is shown in Figure 1. Depending on the type of data, raw or dat, each pixel is recorded at 32 or 16 bits of data. The exact number of rows and columns is stored in a file with the extension hdr that contains all the typical header information. This is information relating to a particular frequency of the spectral range, type of data storage, sensor type, and others. For the analysed data, the image resolution *M* × *N* was varied in the range from 15 × 1312 to 899 × 1312 pixels. The range of changes was strictly dependent on the scan area. This area was limited mainly due to image acquisition time of 12 seconds for the registration of the spectrum at the maximum resolution and in the full range. A dynamic error related to the possible displacement of the scan area during measurements was minimized by mechanical stops and skin area orientation ensuring patient’s comfort.

The images *L*_*GRAY*_(*m*,*n*,*k*), where *m*-row, *n*-column and *k*–the next wavelength *λ* (*k*∈(*1*,*K*)), read from the files with the extension dat or raw were further filtered. For each image in the sequence, a median filter with a mask *h* sized *M*_*h*_ × *N*_*h*_ = 3 × 3 pixels was used. The mask size was dependent on the amount of pollution and the level of noise. In the case of recorded images, the noise and artefacts did not exceed the size of 2 pixels per one cluster. For this reason, a sufficient filter mask size was 3 × 3 pixels. In this way, the noise-free image *L*_*M*_(*m*,*n*,*k*) was subjected to calibration.

### Calibration

**Figure 2:**
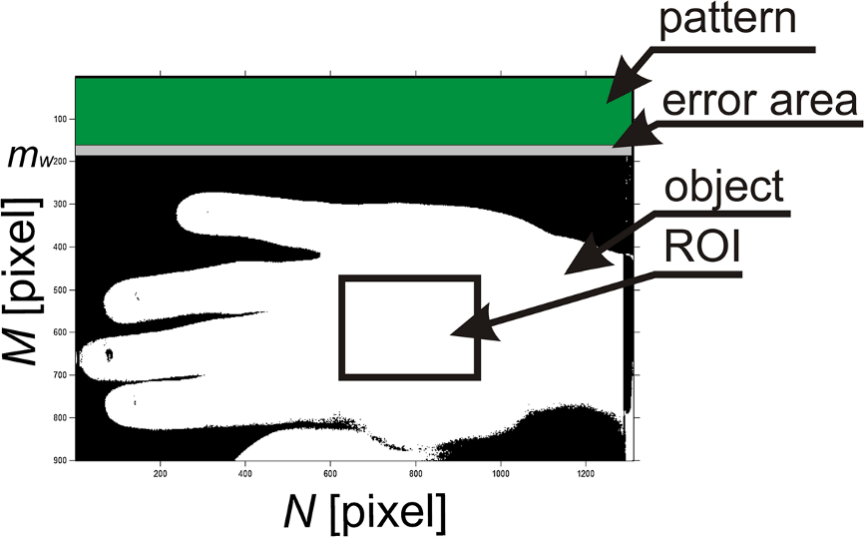
**Location of the pattern in the image during calibration.** The pattern in the form of a white stripe sized 20 × 400 mm, was placed in the upper part of the image. For the set distance from the camera lens it was equivalent to the number of rows *m*_*w*_ = 80 ± 5. The area of uncertainty is highlighted in grey while the pattern area in green. The object, namely the hand, is shown in white.

The acquired images *L*_*M*_(*m*,*n*,*k*) are not calibrated. Calibration involves referring each pixel of the registered skin area to the white pattern [32–34]. For the registered cases, the pattern was a white stripe placed at the top- Figure 2. Automatic detection of the pattern position was implemented in the proposed algorithm. It concerned recognition of one of the pattern contours using information about the brightness gradient of adjacent pixels for each column, i.e.: 1

for *m*∈(1,*N*-1) where *p*_*r*_ is a binarization threshold determined automatically according to Otsu’s formula [35].

The pattern in the form of a white stripe was 20 × 400 mm, which was equivalent to the number of rows *m*_*w*_ = 80 ± 5 for its set distance from the camera lens. The value of ±5 pixels is associated with a possible image shift or rotation. The number of columns was covered by the pattern in its entirety. Therefore, the searched pattern boundary contour was designated as:
2

On this basis, average brightness for each column is calculated, i.e.:
3

**Figure 3:**
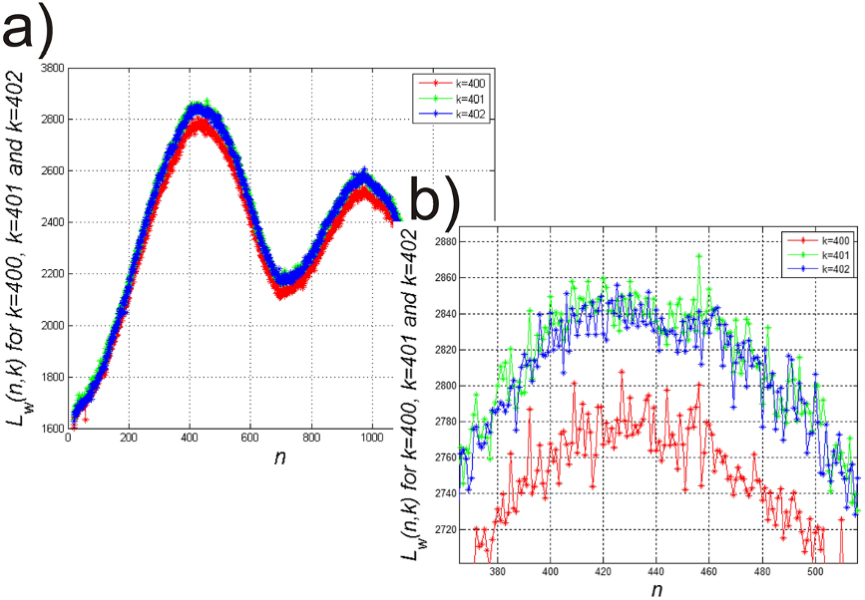
**Graph of changes in average brightness values for each column of the pattern.** The image **a)** and its zoom **b)** show the differences in average brightness values. The image must be calibrated with respect to these changes. These values *L*_*w*_(*n*,*k*) are shown for the sample *k* = 400, 401 and 402. For each value *k*, calibration must be performed independently.

Examples of graphs of *L*_*w*_(*n*,*k*) for *k* = 400, 401 and 402 are shown in Figure 3. The image Figure 3 a) and its zoom Figure 3 b) show the differences in average brightness values. The image must be calibrated with respect to these changes. For each value *k*, calibration must be performed independently. Calibration of individual images is carried out as:
4

for:

In the case of pixels which exceed the value “1”, adjustment is necessary:
5

The image *L*_*K*_(*m*,*n*,*k*) having brightness values in the range from 0 to 1 is further subjected to the next processing steps.

### Image processing

**Figure 4:**
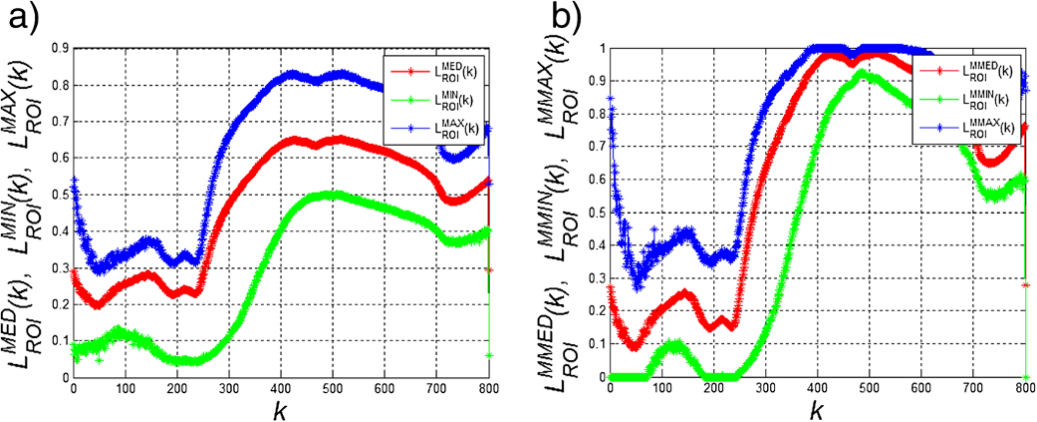
**Graph of changes in the average brightness value in the ROI for** ***k*** **of these images in a series.** The graph **a)** shows changes in the average , minimum  and maximum value  for individual *k*-th images in the sequence. These values are calculated in the ROI covering a sample area of the human hand. The graph **b)** shows changes in the average , minimum  and maximum value  for individual *k*-th images in the sequence but after normalization described in the text.

The input image *L*_*K*_(*m*,*n*,*k*) after calibration is the basis for the segmentation process. For this purpose, a sample diagram of brightness changes in a sample ROI was made for the human skin which mainly consists of water, melanin and haemoglobin. The results for each *k*-th image (at different wavelengths) are shown in Figure 4 a), i.e.:

678

The ROI was associated with the hand area shown in Figure 2 and included the range *M*_*ROI*_ × *N*_*ROI*_ = 150 × 150 pixels. In this range, the values , which are the mean, minimum and maximum values of brightness changes in the ROI respectively, were calculated. The results obtained shown in Figure 4 a) are also dependent on the individual variability of patients and the method of lighting and setting the camera angle relative to the patient. The influence of these elements on the result is revealed by the shift of curves shown in Figure 4 a) up or down, which increases or decreases the mean brightness value. Therefore, normalization performed for the entire image sequence with respect to changes for *k*-th images is necessary, i.e.:
9

for 

For the images *L*_*O*_(*m*,*n*,*k*) modified in this way the obtained results of the mean, minimum and maximum values also change in the same sample ROI, i.e.: . The results obtained are shown in Figure 4b. The normalized images *L*_*O*_(*m*,*n*,*k*) also enable automatic segmentation in accordance with the reference curve of melanin and haemoglobin content for each wavelength. The reference content of melanin and haemoglobin can be acquired from external sources, for example from literature data [36], or on the basis of the selected ROI. In the latter case, the result will be as follows - image *L*_*D*_(*m*,*n*), i.e.:
10

**Figure 5:**
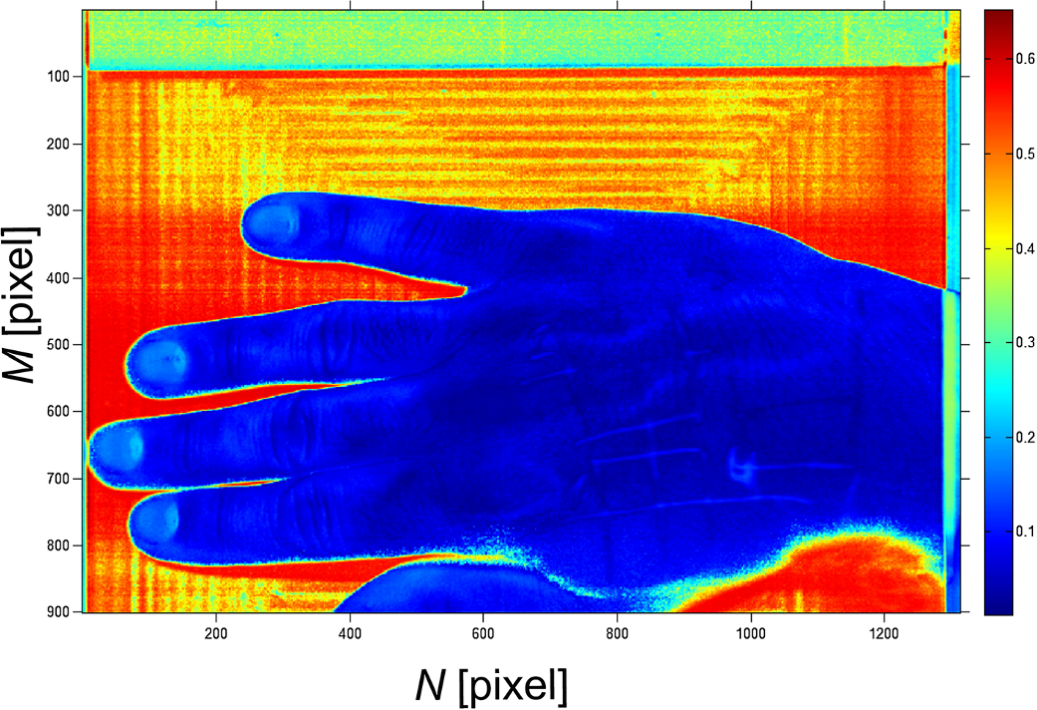
**Image** ***L***_***D***_ **(** ***m*** **,** ***n*** **).** The image *L*_*D*_(*m*,*n*) results from the performed analysis and precedes proper segmentation. Artificial colour palette highlights the individual pixel values. Each pixel is the average difference value with respect to the pattern. For the value below about 0.2 of pixel brightness, skin areas are visible. This fact will be used for further analysis.

Therefore, the image *L*_*D*_(*m*,*n*) contains information about the average error- Figure 5. It is calculated for individual pixels relative to the reference waveform . On this basis, binarization-based segmentation can be performed for the binarization threshold *p*_*w*_ designated manually or automatically (the afore-mentioned Otsu formula [35]). From a practical point of view, the effect of the binarization threshold selection (provided manually) on the segmentation results obtained is of interest. For this purpose, the impact of changes in the threshold *p*_*w*_ on the changes in the surface area *A*(*p*_*w*_) of the segmented object was investigated, i.e.:
11

where:
12

**Figure 6:**
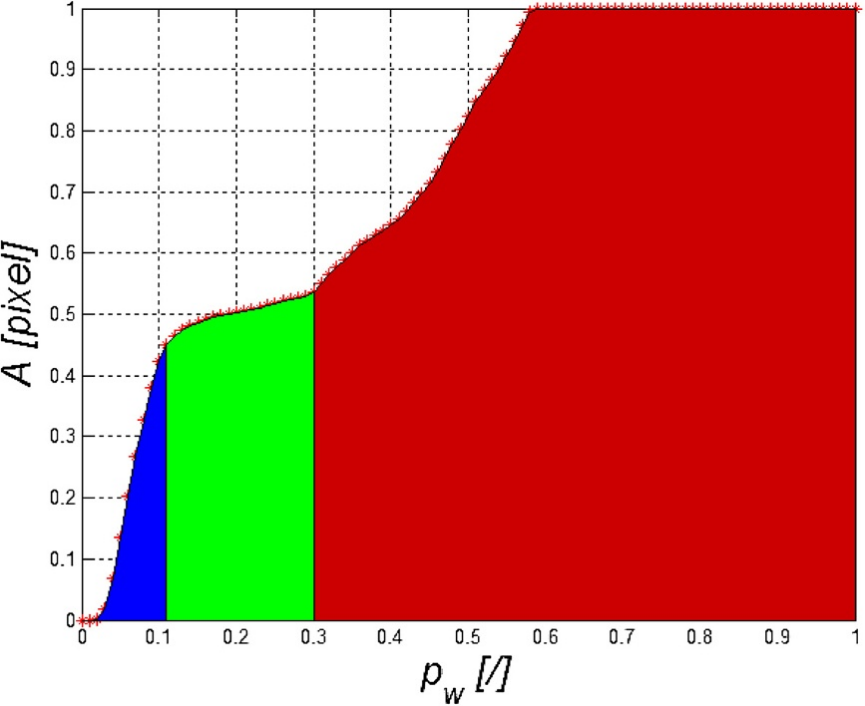
**Graph of changes in the surface area** ***A*** **(** ***p***_***w***_ **) for different binarization thresholds** ***p***_***w***_ **.** The colours indicate characteristic areas: blue refers to the area of too low threshold value (the object surface is too small), green highlights the area of changes in binarization threshold giving the best segmentation results whereas red indicates too high binarization threshold (the resultant segmented object is too large).

The results obtained are shown in Figure 6. The area optimal from the point of view of the segmentation results is marked in green. The term “optimal” refers to such a fragment of the curve *A*(*p*_*w*_) which is a flat area. For the threshold *p*_*w*_ = 0.2 ± 0.9, the segmentation result is correct. This fact was proven when comparing it with the result obtained by an expert relying on manual marking (gold standard). The error is here defined as:
13

where *A*_*Z*_ is the surface area resulting from the expert’s work.

The results obtained from equation (13) it was assumed that the results obtained by the expert are repetitive and do not differ from results obtained by other experts. Not in every case, however, it must be so. The exact considerations presented in [37–39].

**Figure 7:**
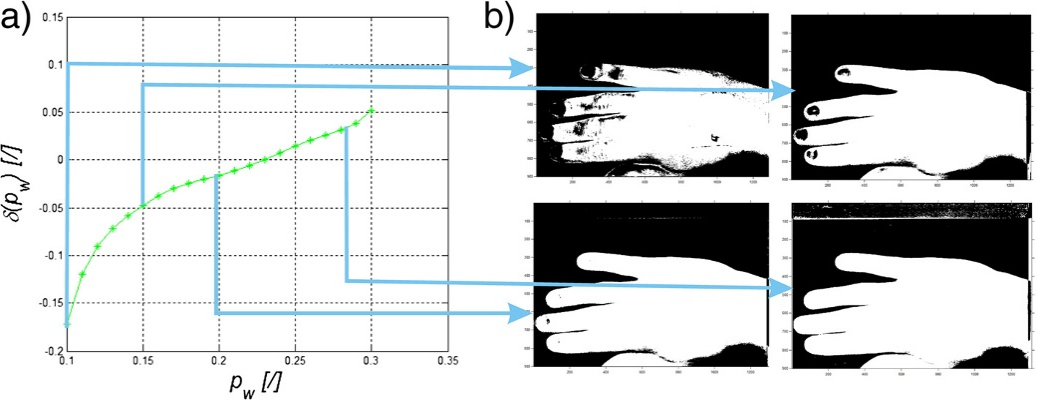
**Graph of changes in the error** ***δ***_***w***_ **for varying binarization thresholds** ***p***_***w***_ **.** In the presented graph **a)** the smallest error (*δ*_*w*_ ≈ 0) is obtained for the threshold *p*_*w*_ = 0.23. Part **b)** shows some subsequent results of patient’s hand area binarization. The selected binarization results are for sample thresholds *p*_*w*_∈{0.1, 0.15. 0.2, 0.27}.

The results of changes in the error *δ*_*w*_ for varying *p*_*w*_ (*p*_*w*_∈(0.1, 0.3)) are shown in Figure 7a). From the presented graph and for the case under consideration, the smallest error (*δ*_*w*_ ≈ 0) is obtained for *p*_*w*_ = 0.23. For automatic selection of the binarization threshold (Otsu’s formula), the error is 9%. Sample binarization results are shown in Figure 7b for the thresholds *p*_*w*_∈{0.1, 0.15. 0.2, 0.27}. Visual assessment gives the best results for *p*_*w*_ = 0.23. However, it should be noted here that the adopted binarization threshold values are the acceptable standard deviation of the reference distribution of the skin spectrum from the measured pixels (formula (10)). In general, for any image containing the human skin, the following approaches are possible:

- automatic selection of the binarization threshold according to the Otsu’s formula – it enables to obtain a binary image of the object,
- manual selection of the binarization threshold *p*_*w*_ dependent on the acceptable tolerance of individual pixels of the image relative to the reference waveform  - 1% tolerance is *p*_*w*_ = 0.01, 10% tolerance is *p*_*w*_ = 0.1 respectively, etc.,
- automatic selection of the binarization threshold *p*_*w*_ depending on the location of the ‘flat’ area (Figure 6).

**Figure 8:**
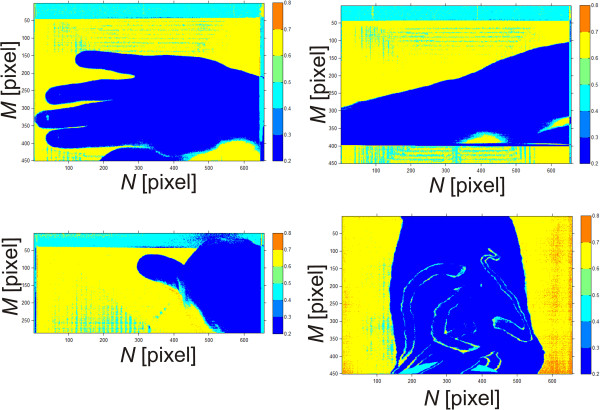
**Sequence of images** ***L***_***DB***_ **(** ***m*** **,** ***n*** **) for** ***p***_***w***_ **∈(0,1).** The presented image sequence is for artificial colour palette for 0.2 step. Sequences for 4 different images are shown: **a)** image of the hand, **b)** forearm, **c)** thumb and **d)** tattoo. In all cases, the results obtained for *p*_*w*_ = 0.4 covered the object containing the human skin.

Depending on the desired end result, one of the above methods is selected by an operator. Figure 8 shows a sequence of images *L*_*DB*_(*m*,*n*) for *p*_*w*_∈(0,1) changed with 0.2 step from the area of the hand, forearm, finger (thumb) and tattoo.

## Results

The results obtained, namely the images *L*_*DB*_(*m*,*n*) and *L*_*D*_(*m*,*n*) as well as *L*_*O*_(*m*,*n*,*k*), enable any segmentation of objects in the image. The division into the object and background, most commonly used in practice, can be implemented on the basis of the equation (10). The division into three areas (I, II and III) can be implemented in a similar way. This type of result was achieved by modifying the equation (10) to the following form, for example for the area *I*:
14

where *i* – subsequent image pixels, i.e. *i*∈(*1*, *M∙N*), – reference values for the spectrum characteristic for the object *I.*

**Figure 9:**
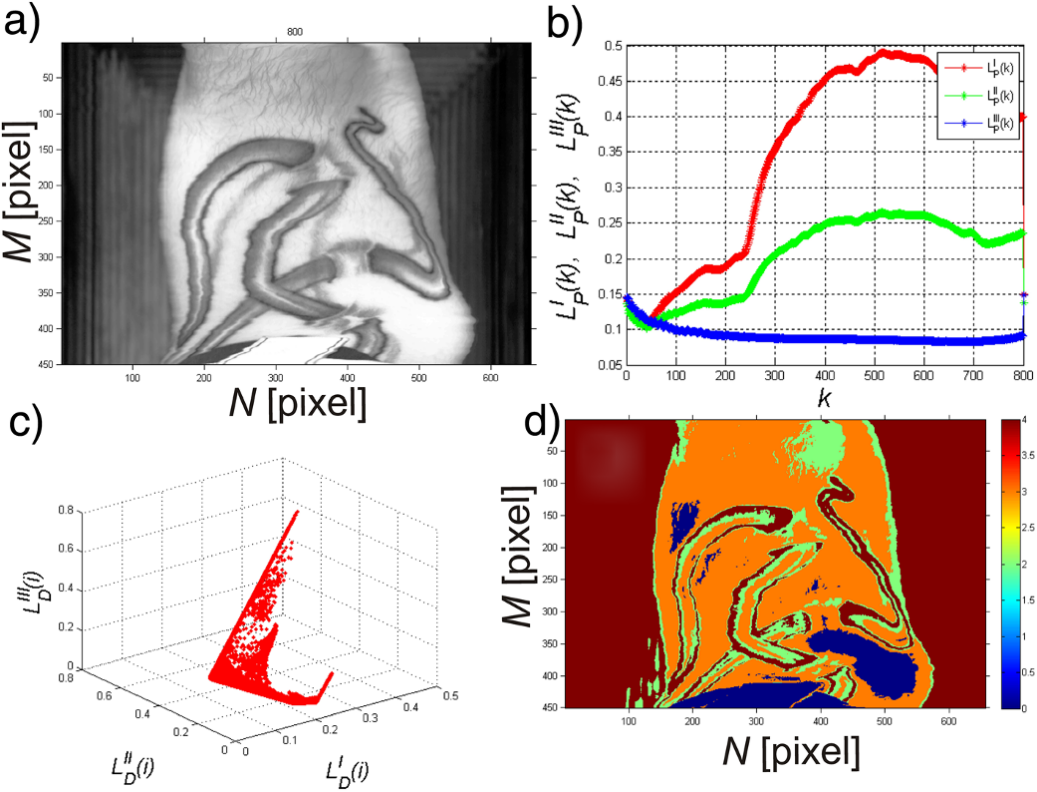
**Results obtained for a sample tattoo image.** Figure **a)** shows a sample hyperspectral image for *k* = 300. Figure **b)** shows reference waveforms obtained based on the spread spectrum curves known from the literature which are the pattern. Figure **c)** shows a graph  for each *i*-th pixel. Figure **d)** shows the results of segmentation performed based on the algorithm described in the paper.

The thus designated absolute difference values  are the basis for the segmentation of individual objects. Figure 9 b) shows sample reference waveforms obtained based on the spread spectrum curves known from the literature which are the pattern [11, 36]. Figure 9 c) shows the graph  for each *i*-th pixel. Segmentation in this case concerned the separation of the tattoo area in Figure 8 c) (Figure 9 a) from the skin and image background - for the adopted accuracy value of *p*_*w*_ = 0.2 for all objects and the background. The results of the comparison of the areas selected by an expert (of the tattoo, skin and background) with the results obtained using the presented algorithm is shown in Table 1. In addition to the areas of tattoo, the skin and the background, there remains an area (19’869 pixels - 6.7%) which is not assigned to any of the objects. This area is not classified. When increasing accuracy, namely the value *p*_*w*_, the surface areas of individual areas increase, which may lead to the overlap of adjacent areas. In this case, free, not classified, areas were formed. The highest measurement error (61.5%) is related to the tattoo area. This is because of the absence of additional processing and analysis of the obtained images *L*_*DB*_(*m*,*n*). One of the methods to improve this result involves conditional erosion and dilation. In the case of a symmetrical structural element *SE*(*m*_*SE*_,*n*_*SE*_), the relationships of conditional erosion and dilation are simplified to the following form:
1516

where: *L*_*E*(*C*)_(*m*,*n*) – the resulting binary image after subjecting the image *L*_*DB*_ to conditional erosion,

**Table 1:** **Comparison of segmentation results obtained by an expert with the results obtained from the presented algorithm**

	Tattoo [pixels]	Skin [pixels]	Background [pixels]	Non-identified areas [pixels]	Measurement error of tattoo surface area [%]	Measurement error of skin surface area [%]	Measurement error of background surface area [%]
Expert	19411 (6.6% of the whole image)	136780 (46.3% of the whole image)	139009 (47.1% of the whole image)	0 (0%)	61.5%	5.1%	2.6%
Algorithm	31356 (10.6% of the whole image)	101312 (34.4% of the whole image)	142663 (48.3% of the whole image)	19869 (6.7%)			

*L*_*D*(*C*)_(*m*,*n*) – the resulting binary image after subjecting the image *L*_*DB*_ to conditional dilation,

*p*_*we*_ – the constant that determines erosion effectiveness,

*p*_*wd*_ – the constant that determines dilation effectiveness,

*p*_*mn*_(*m*,*n*) – the threshold dependent on the coordinates *m*, *n*,

*s*_*re*_ – the mean value of the analysed area for erosion,

*s*_*rd*_ – the mean value of the analysed area for dilation.

The mean values *s*_*re*_, *s*_*rd*_, for erosion and dilation respectively, were calculated form the following equations: 1718

The constants *p*_*we*_ and *p*_*wd*_, which determine the effectiveness of erosion and dilation respectively, take values from the range (0,1), i.e.: *p*_*we*_∈(0,1) and *p*_*wd*_∈(0,1). The values from this range arise directly from the condition of the left side of inequality (15), i.e.:
19

**Figure 10:**
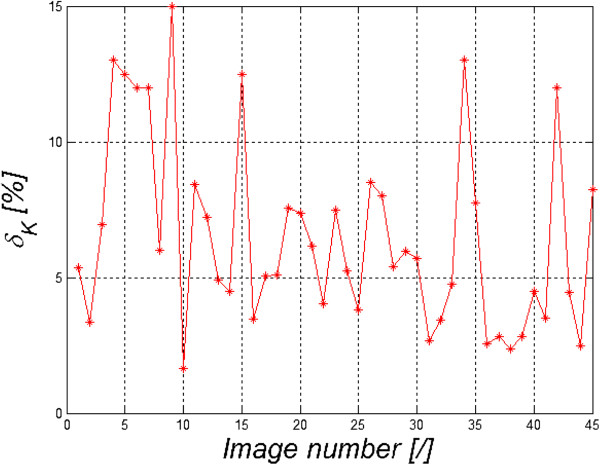
**Maximum error values obtained for the analysed 45 hyperspectral images (36’000 2D images).** These errors are calculated as a result of the comparison of surface area calculations for individual images containing the human skin with segmentation performed by an expert. The sequence of images on the x-axis is random.

The values of *p*_*nm*_∈(0,1), whereas the values (1–*p*_*we*_) should be non-negative in the range from 0 to 2. The values (1–*p*_*we*_) and (1 + *p*_*wd*_) for *p*_*we*_ *= p*_*wd*_ = 0 are equal to 1, which means high intensity of conditional operations. For the other values of thresholds *p*_*we*_ and *p*_*wd*_, for example, for *p*_*we*_ *= p*_*wd*_ = 1, there is a complete lack of effectiveness of the erosion operation and significant effectiveness of dilation. In the present case *p*_*mn*_(*m*,*n*) *=* const and is independent of the location (*p*_*mn*_ ≠ *f*(*m*,*n*)). The shape of the structural element SE in all these relationships was adopted as round sized 5 × 5 pixels due to the shape and size of the smallest objects corrected. These properties of conditional erosion and dilation enable to obtain effective correction of the surface of individual areas - in this case the tattoo, skin and background. This adjustment involves sequential implementation of conditional erosion and dilatation (in this case five times) for the images *L*_*DB*_(*m*,*n*) and *L*_*D*_(*m*,*n*). The binary image *L*_*DBC*_(*m*,*n*), adjusted in this way, enables to obtain much better results - Table 2. The pixels not allocated to any of the areas were eliminated owing to the operations of conditional erosion and dilation. However, the skin area measurement error increased and in this case amounted to 12.9%. This error is closely dependent on the amount of segmented image areas, on the type and differences in the spectra for individual areas. Finally, the results obtained have a segmentation error of less than 13% compared to the work of the expert. Figure 10 shows segmentation errors for 45 hyperspectral image sequences, 36’000 2D images, analysed with the present method. The average value of the described method error *δ*_*K*_ fluctuates around 9% (the maximum value is 23%, minimum - 1%). These results are in the next section compared with the results obtained by other authors.

**Table 2:** **Comparison of segmentation results obtained by an expert with the results obtained from the presented algorithm after adjustment with conditional erosion and dilation**

	Tattoo [pixels]	Skin [pixels]	Background [pixels]	Non-identified areas [pixels]	Measurement error of tattoo surface area [%]	Measurement error of skin surface area [%]	Measurement error of background surface area [%]
Algorithm	19066 (6.5%)	138365 (46.9%)	138114 (46.8%)	0 (0%)	1.8%	12.9%	1%

## Comparison with other authors’ results

Hyperspectral imaging is the subject of many works on spectrum measurement and its analysis. In many studies, also simple functions related to image analysis and processing are used. Compared to conventional monochrome images, hyperspectral imaging contains much more information. This means that typical known methods of image analysis and processing must be generalized or profiled to their particular application. Among other things, the fact of having information on the complete spectrum in a specified range results in the possibility of performing more accurate segmentation than during typical segmentation. The analysis itself in the known reference works, however, usually refers to the analysis of the manually selected ROI, e.g. in [28]. There are many other areas of medicine which use similar manual or semi-automatic selection of the region of interest. These are, for example, the areas mentioned in [36] - vibrational hyperspectral imaging Filik J. [40], laparoscopic digital light processing – Olweny EO [41], blood stains at the crime scene – Edelman G. [42], prostate cancer detection – Akbari H. [43], histopathological examination of excised tissue - Vasefi F, diabetic foot ulcer - Yudovsky D [44], cancer detection - Akbari H [45], and others. In medicine, the use of hyperspectral imaging to assess the creation time of a bruise is also known – Stam B. [46]. The inaccuracy found is 2.3% for fresh bruises and 3 to 24% for bruises up to 3 days old. In conclusion, colour inhomogeneity of bruises can be used to determine their age. The experiment results in the work of Li Q [28] show that the hyperspectral based method has the potential to identify the spinal nerve more accurately than the traditional method as the new method contains both the spectral and spatial information on nerve sections. A strong resemblance of hyperspectral imaging to monochrome imaging means that typical methods such as morphological operations can be applied here. They are used in the classification of different types of artefacts visible in images [1, 13, 15] in SVM (support vector machines) [14], Gauss-Markov model [16] or wavelet analysis [18]. For example, in the work of Dicker et al. [31] spectral library was generated with 12 unique spectra that were used to classify specimens where sample preparation was varied. The work of Benediktsson J. et al. [11] presents results for the sequential use of morphological opening and closing for the increased size of the structural element. This methodology is similar to the use of conditional erosion and dilation, as in [13] and G. Rellier’s work [17]. These known methods of image analysis and processing do not enable fully automatic segmentation of the skin area, especially in conjunction with the prior automatic calibration.

## Critical summary

**Figure 11:**
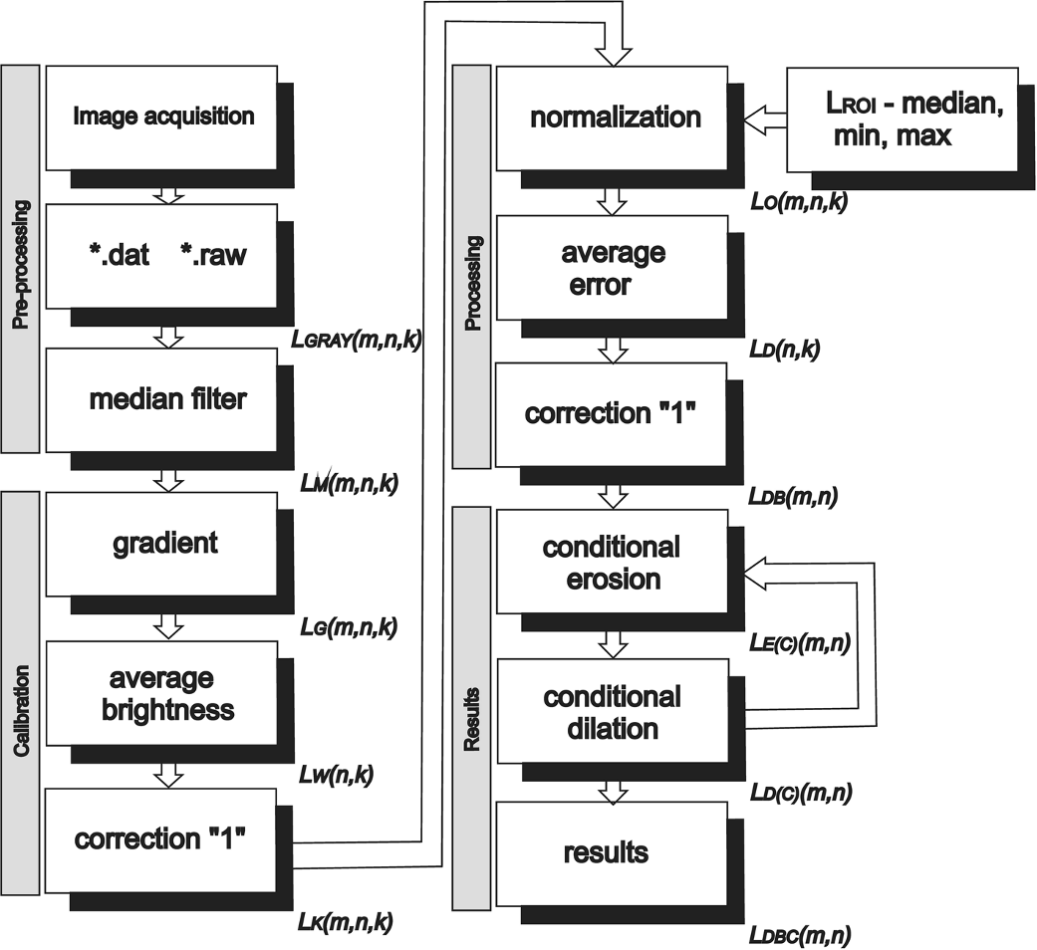
**Block diagram of the proposed algorithm for spectral image analysis and processing.** The sequence of images is converted from *.dat or *.raw format to a three-dimensional image matrix, and then there follows median filtration, automatic image calibration, segmentation of the skin areas and final analysis**.**

The paper presents the method of calibration and segmentation of selected skin areas in hyperspectral imaging. The characteristics of the method described, whose block diagram is shown in Figure 11, include:

- the possibility of automatic calibration with the use of a white pattern placed in the upper part of the image (in the present case, the pattern was rectangular),
- the possibility of automatic segmentation of the skin area,
- the possibility of automatic segmentation of several areas, including those visible in the same image on the basis of ray spectrum,
- measurement repeatability owing to full elimination of operator’s intervention in the study,
- the possibility of any quantitative (not qualitative) assessment of the results obtained - the surface area of the segmented areas and others,
- time analysis of a sequence of images does not exceed 10 s using Intel Core i5 CPU M460 @2.5GHz 4GB RAM.

The presented method can be extended to:

- full analysis of the expert’s impact on the result. In the described case, there was one expert. In future studies, repeatability of the expert’s work and possible differences in the work of several experts should be verified.
- analysis of the impact of the hyperspectral camera operator on the result. Individual operator’s habits, placing an object on the stage may be vital for the results obtained.
- analysis of the portability of this algorithm to other medical institutions. The impact of image resolution on the result, the impact of specific settings of the algorithm - its parameters.

Therefore, the discussed algorithm for image analysis and processing does not fully cover the issue. In terms of application, the techniques from spectral methods [47–49], analysis of microscopic images [50, 51] and others [52–59] can also be used. This type of analysis will be carried out by the authors in future studies in this area.

## Abbreviations

std: Standard deviation
ROI: Region of interest.
